# Supplementary material for: Composite Nanoarchitectonics with CoS2 Nanoparticles Embedded in Graphene Sheets for an Anode for Lithium-Ion Batteries
Source: Nanomaterials (Basel). 2022 Feb 21;12(4):724. doi: 10.3390/nano12040724 (PMC8875400; doi:10.3390/nano12040724)
Supplement: Supplementary file 1 [file nanomaterials-12-00724-s001.zip › nanomaterials-1539209-supplementary.pdf]

nanomaterials

Supplementary Materials

Composite Nanoarchitectonics with CoS_2 Nanoparticles Embedded in Graphene Sheets for an Anode for Lithium-Ion Batteries

Tongjun Li [1,2,3,4], Hongyu Dong [2,3,4,*], Zhenpu Shi [1,2], Hongyun Yue [2,3,4], Yanhong Yin [2,3], Xiangnan Li [1,2], Huishuang Zhang [1,2,4], Xianli Wu [5], Baojun Li [5] and Shuting Yang [1,2,3,4,*]

1 School of Physics, Henan Normal University, Xinxiang 453007, China; tongjunxiaoyu@163.com (T.L.); zhenpushi@foxmail.com (Z.S.); lxn506@163.com (X.L.); zhanghuishuang0000@163.com (H.Z.)
2 School of Chemistry and Chemical Engineering, Henan Normal University, Xinxiang 453007, China; yuehongyun@foxmail.com (H.Y.); yinyh@163.com (Y.Y.)
3 National and Local Joint Engineering Laboratory of Motive Power and Key Materials, Xinxiang 453007, China
4 Collaborative Innovation Center of Henan Province for Motive Power and Key Materials, Xinxiang 453007, China
5 College of Chemistry, Zhengzhou University, Zhengzhou 453000, China; wuxianli@zzu.edu.cn (X.W.); lbjfcl@zzu.edu.cn (B.L.)
* Correspondence: donghy373@163.com (H.D.); shutingyang@foxmail.com (S.Y.); Fax/Tel.: +86-373-332-6439 (S.Y.)

Fabrication of the Half Cells

Li-battery performance was determined using CR 2032 type coin cells assembled in an argon–filled glove box (MBRAUN). The prepared active material, the conductive agent acetylene black and the binder polyvinylidene fluoride (PVDF) were mixed evenly according to the mass ratio of 8:1:1 and adjusted into paste. Coat the copper foil evenly with a coating device, dry it at room temperature, and dry it at 60 °C for 12 h in a vacuum drying oven. The dry electrode was cut into 12 mm diameter discs and pressed with 6 MPa pressure to prepare the working electrode for use (the area mass load of the electrode was 1.8 mg/cm^2). With lithium metal as counterelectrode and reference electrode, the test cell was assembled in a glove box filled with argon gas using a polypropylene diaphragm (PP) and 1 mol/L $LiPF_6$ (DMC:EC = 1:1, volume ratio) as electrolyte. Under different rates, CT2001A blue battery test system was used to conduct constant current charge and discharge test in the voltage range of 0.05–3 V. The test cell is tested with Cyclic Voltammetry (CV) and electrochemical impedance spectroscopy (EIS) on an electrochemical workstation, with the frequency range of AC impedance measurements being 10^{-1}–10^{-5} Hz.

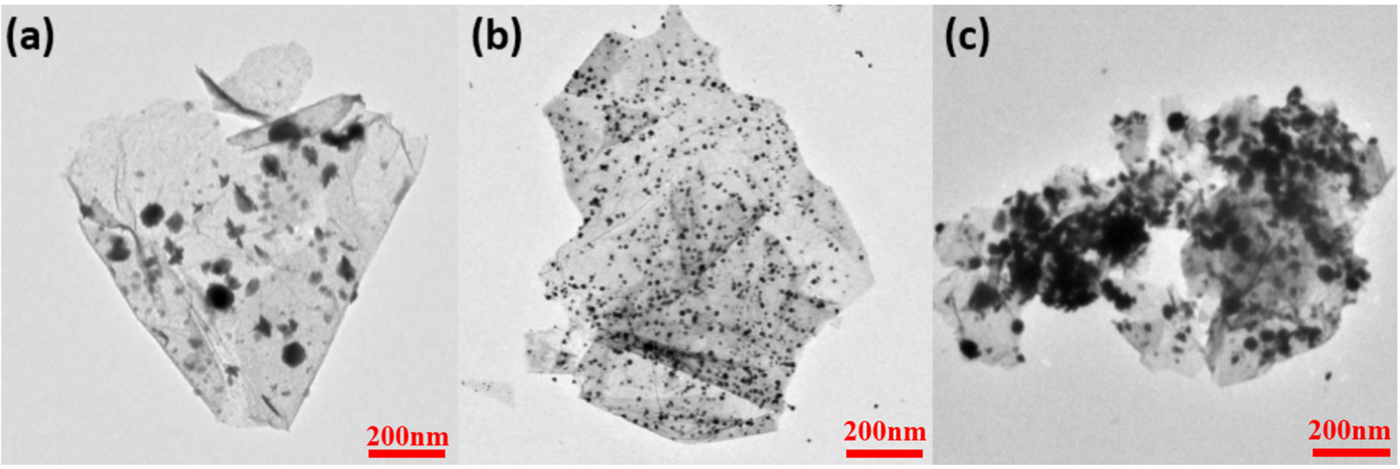

Figure S1. (**a**–**c**). The quality of GO added is the TEM image corresponding to 1:2:3.

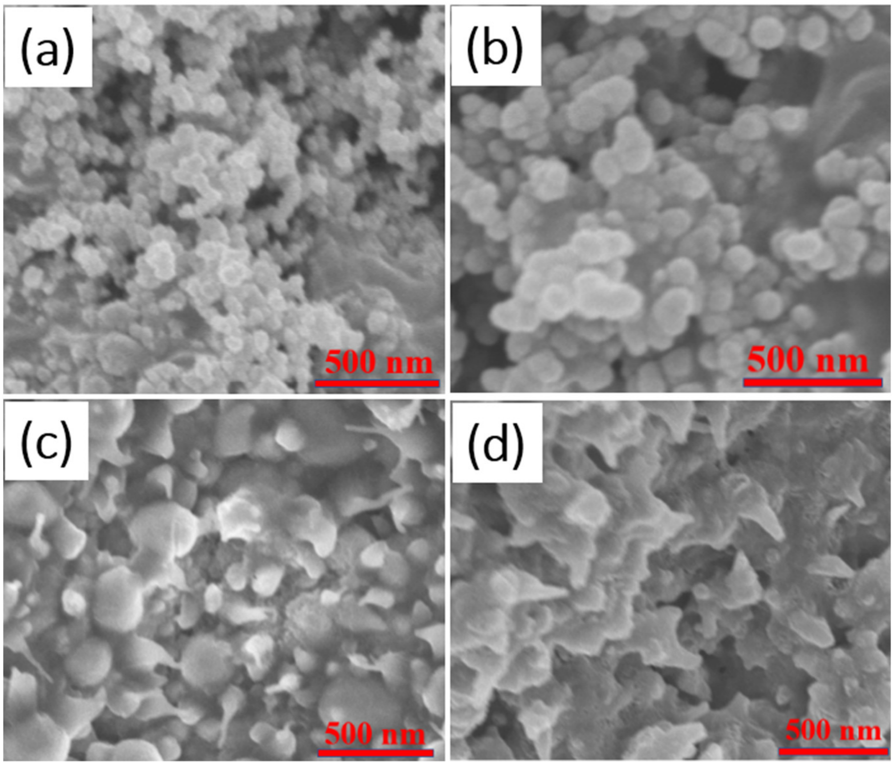

Figure S2. (**a**) CoS_2@rGO material morphology after 50 cycles; (**b**) Morphology after 100 cycles; (**c**) Morphology after 150 cycles; (**d**) Morphology after 500 cycles.

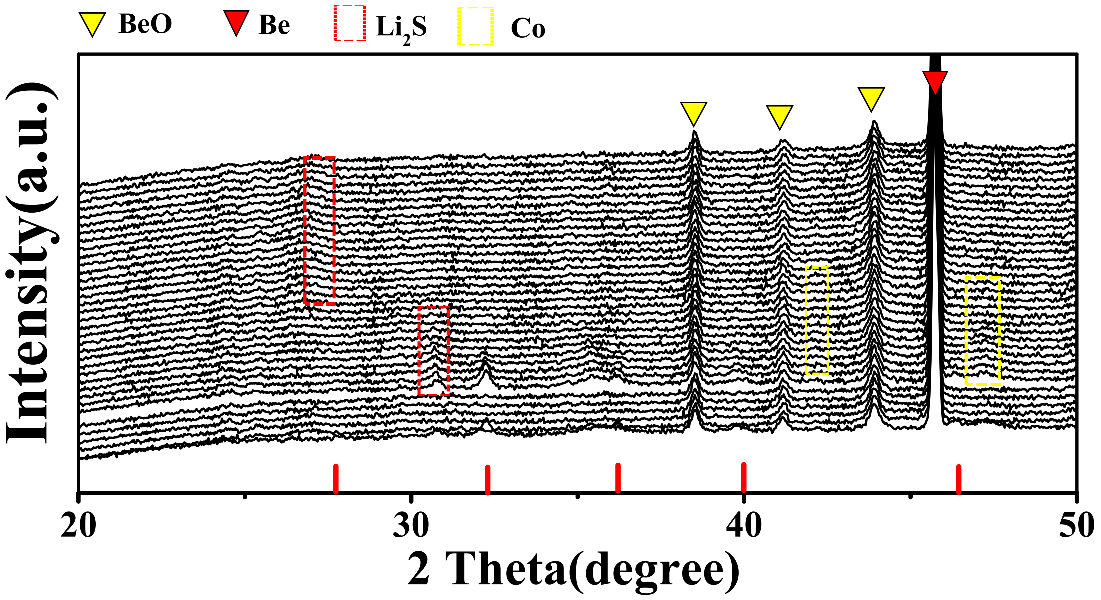

Figure S3. show an expanded region from 20°. to 50°.

Table S1. The experimental materials and drugs.

Name of Reagent	Specifications and Models	The Manufacturers
GO	-	Self-made
C	AR	Beijing Bailingwei Technology Co., LTD
H_2SO_4	AR	Luoyang Chemical Reagent Factory
HNO_3	AR	Luoyang Chemical Reagent Factory
H_2O_2	AR	Tianjin Deen Chemical Reagent Co., LTD
$CoCl_2{\cdot}6H_2O$	GR	Tianjin Deen Chemical Reagent Co., LTD
$NaBH_4$	AR	Aladdin Co. LTD
$C_4H_9NO_2S$	GR	Aladdin Co. LTD
$KMnO_4$	GR	Aladdin Co. LTD
$NaNO_4$	GR	Aladdin Co. LTD
C_2H_5OH	AR	Beijing Bailingwei Technology Co., LTD
NMP	AR	Henan Li Dynamic Power Supply Co. LTD
DMC	AR	Henan Li Dynamic Power Supply Co. LTD
PVDF	AR	Henan Li Dynamic Power Supply Co. LTD
PP	AR	Henan Huarui High-tech Materials Co., LTD
$LiPF_6$	AR	Henan Huarui High-tech Materials Co., LTD

Table S2. Electrochemical performance comparison of the as-prepared CoS_2@rGO anode with other previously reported CoS_2-based anodes in LIBs.

Types of Materials	Current Density (A g^{-1})	Cycle Number (N)	Specific Capacity (mAh g^{-1})	Ref.
CoS_2/MCF	1.0	300	630	[3]
CoS_2/rGO	0.1/1.0	50/150	648/458	[41]
CoS_2/CNTs	0.1	100	568	[42]
$NiCo_2S_4$@NiCo2S_4	0.2	100	334	[6]
Nano-sized CoS_2	0.1	50	302	[7]
3D spongy CoS_2/C	0.5	120	610	[8]
Cross-nanoflower CoS_2	0.5	100	650	[43]
CoS_2@rGO	0.2/3.0	100/305	739/288	This work
